# Time-course changes in fingernail cortisol levels during pregnancy and postpartum

**DOI:** 10.1038/s41598-024-51650-2

**Published:** 2024-01-11

**Authors:** Shuhei Izawa, Mikiko Kawasaki, Nagisa Sugaya, Shusaku Nomura

**Affiliations:** 1 Occupational Stress and Health Management Research Group, National Institute of Occupational Safety and Health, Kawasaki, Japan; 2 Graduate School of Medicine, International University of Health and Welfare, Tokyo, Japan; 3 Japanese Red Cross Kyushu International College of Nursing, Munakata, Japan; 4 Faculty of Engineering, Nagaoka University of Technology, Nagaoka, Japan

**Keywords:** Endocrinology, Biomarkers, Psychology

## Abstract

The cortisol level in fingernails can reflect the cumulative hormones produced in the body several months prior. However, previous studies have only demonstrated the cross-sectional associations of fingernail cortisol with salivary or hair cortisol, and not longitudinal changes in fingernail cortisol in situations where cortisol levels in the body could be expected to change. Therefore, this study focused on pregnancy as a model for changes in cortisol levels over a prolonged period of time, and investigating the time courses of fingernail cortisol during pregnancy and the postpartum period. We collected nail samples from 30 healthy women during pregnancy and 12 months postpartum to measure the cortisol levels in the nail. Results showed that cortisol levels in fingernail clippings increased from 1 month before childbirth to 4 months postpartum, with the levels peaking at 2 months postpartum. Additionally, we found higher cortisol levels in fingernail clippings in primiparas than in those of multiparas. The time course of fingernail cortisol levels could replicate the longitudinal changes in cortisol in the body, and differences between multiparas and primiparas seemed to be biologically plausible, which could support the concept of fingernail cortisol as a retrospective index of hormone production.

## Introduction

Cortisol levels in blood and saliva are well known to increase in response to acute psychosocial stress^1^. Over the past two decades, cortisol levels in hair samples have been investigated as an index of the cumulative hormone^2^. Cortisol enters hair at the level of the medulla of the hair shaft via passive diffusion from blood during hair follicle formation. Scalp hair grows at an average rate of 1 cm/month, and cortisol secreted in the past month can be measured from the 1 cm segment closest to the scalp. Although saliva and blood samples have revealed instantaneous hormone values, hair samples provide an index of cumulative hormone exposure over a longer period.

Cortisol contained in nail samples has attracted attention as a possible index of cumulative hormone^3,4^. Compared to hair samples, nail clippings are easily collected by the participants themselves, and the participants are unlikely to hesitate to submit the samples because these could usually be discarded in their daily life. Endogenous hormones passively diffuse from the capillaries into the nail matrix and are incorporated into keratin during nail formation^5^. Fingernails grow at an average rate of 3 mm/month^6^, and 3–5 months are generally required for the nail to fully extend from the matrix^7^; therefore, it was considered that cortisol contained in fingernail clippings could represent the hormone levels from several months prior^8^. This concept has been partially supported by previous studies, in which higher fingernail cortisol levels were associated with major depressive disorder^9^, onset of an acute coronary syndrome^10^, and stressful experiences in the past^11–13^.

However, the concept of fingernail cortisol as a retrospective index of hormone production has not yet been fully investigated. From the perspective of validity of fingernail cortisol, two previous studies demonstrated that cortisol levels in fingernail clippings were retrospectively correlated with cortisol levels in hair and saliva samples with a time lag of 2–5 months in middle-aged workers^14^ and young adults^15^. These studies demonstrated a correlation between cortisol levels in the body and fingernails at the inter-individual level, but no evidence that intra-individual changes in body cortisol could be reflected in fingernail cortisol levels. No previous studies have investigated intra-individual changes in fingernail cortisol levels in situations where body cortisol can be expected to change. Therefore, further investigation is required to understand the concept of fingernail cortisol.

In this study, we focused on pregnancy as a model for changes in cortisol levels over a prolonged period of time and investigated the time course of fingernail cortisol levels during pregnancy and postpartum. Cortisol levels in the blood gradually increase during pregnancy, peak in the third trimester of pregnancy, and sharply decrease after childbirth (e.g.,^16,17^). This is caused by increased secretion of corticotropin-releasing hormone expressed in the placenta during pregnancy. We hypothesized that the already well-known transition of cortisol in the body of pregnant women, as described above, would be similarly observed in nail cortisol and that the transition in nail cortisol would have a time delay of several months due to nail growth. We also hypothesized that the level of fingernail cortisol would differ between multiparas and primiparas. Compared with women who had given birth before (multiparas), those giving birth for the first time (primiparas) exhibited higher cortisol levels in blood and hair samples during pregnancy (e.g.,^18,19^). Therefore, we expected higher cortisol levels in the fingernail clippings of primiparas collected postpartum. Furthermore, we explored the time-course changes in toenail cortisol during pregnancy and postpartum because only a few studies^20^ have focused on toenail cortisol as a retrospective index of hormone production.

## Results

### Time course of fingernail cortisol

**Figure 1:**
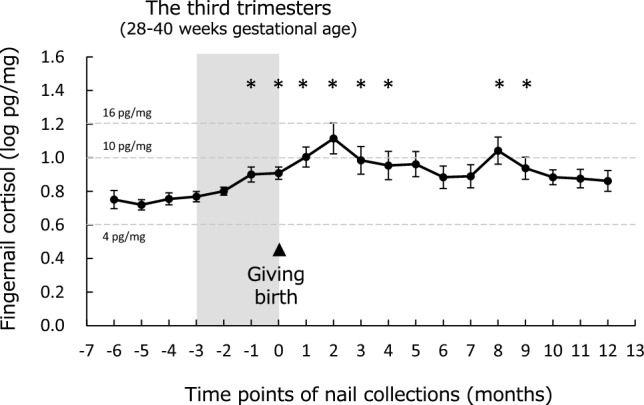
Time course of fingernail cortisol levels during pregnancy and postpartum. Cortisol levels without logarithmic transformation are indicated by dashed lines. * Significant differences from baseline cortisol levels (6 months before giving birth) using the Benjamini–Hochberg adjustment.

Demographic variables of the participants are presented in Table 1. The time courses of fingernail cortisol levels are shown in Fig. 1. Linear mixed models detected significant main effects of time point (F [18.0/28.5] = 4.3, *p* < 0.001). Post-hoc analyses indicated that cortisol levels from 1 month before childbirth to 4 months postpartum, as well as those at eight and 9 months postpartum, were higher than the level at 6 months before childbirth. Linear mixed models adjusted for age, BMI, marital status, employment status, cesarean section, seasons of nail collection, COVID-19 pandemic period, postpartum depression, nail volume, frequency of washing hands using soap, and use of alcohol, or frequency of detergent use were also performed, but the significance of time points and time courses of fingernail cortisol were not significantly altered.

**Table 1:** Demographic data of the participants.

Variables	Means ± standard deviations, N
Age (years)	30.5 ± 4.7
BMI before pregnancy (kg/m^2^)	20.8 ± 2.7
Marital status (married/unmarried)	29/1
Employment status (employee/unemployed)	22/8
History of giving birth (primiparas/multiparas)	18/12
Experience of cesarean section during childbirth (yes/no)	2/28
Season during childbirth (spring/summer/autumn/winter)	9/4/10/7
Giving birth during the COVID-19 pandemic (yes/no)	4/26
Experiencing depression during the 12 months postpartum (yes/no)^a^	14/16
Washing hands using soap (frequency per day) ^b^	8.7 ± 5.2
Washing hands using alcohol (frequency per day)^b^	2.2 ± 2.1
Detergent use without wearing gloves (frequency per day)^b^	3.2 ± 1.3

^a^Postpartum depression was considered experienced if participants exhibited a Japanese version of the Edinburgh Postnatal Depression Scale score ≥ 9 one or more times during the 12 months after childbirth.

^b^Averages over the study period were demonstrated.

### Parity and time course of fingernail cortisol

**Figure 2:**
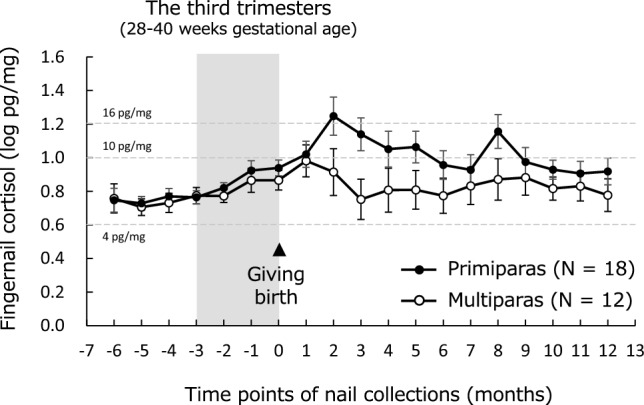
Time course of fingernail cortisol levels during pregnancy and postpartum in primiparas and multiparas**.** Cortisol levels without logarithmic transformation are indicated by dashed lines.

The time courses of fingernail cortisol levels in primiparas and multiparas are shown in Fig. 2. Linear mixed models for fingernail cortisol detected significant main effects of time point (F [18.0/27.6] = 4.3, *p* < 0.001), parity (F [1.0/28.0] = 4.4, *p* = 0.045), and an interaction between time point and parity (F [18.0/27.6] = 2.6, *p* = 0.028). Primiparas exhibited higher fingernail cortisol levels after giving birth. Primiparas were younger than multiparas (t[28] = 2.4, *p* = 0.025); therefore, linear mixed models adjusting for age were further conducted, in which a significant interaction between time point and parity was still detected (F [18.0/27.5] = 2.2, *p* = 0.028). The other demographic variables were comparable between the groups (S1 Table).

### Time course of toenail cortisol

**Figure 3:**
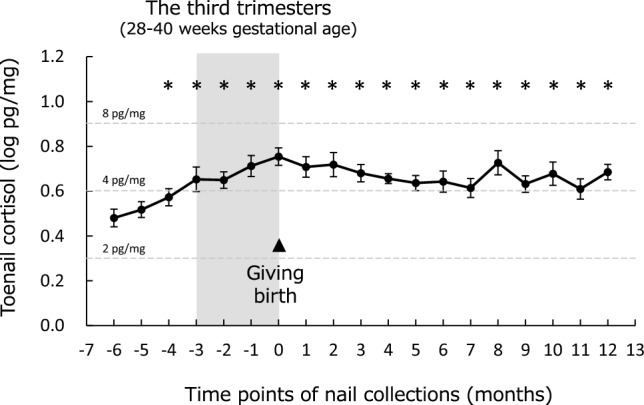
Time course of toenail cortisol during pregnancy and postpartum. Cortisol levels without logarithmic transformation are indicated by dashed lines. * Significant differences from baseline cortisol levels (6 months before giving birth) using the Benjamini–Hochberg adjustment.

The time courses of toenail cortisol levels are shown in Fig. 3. Linear mixed models detected significant main effects of time point (F [18.0/28.3] = 9.2, *p* < 0.001). Post-hoc analyses indicated that cortisol levels from 4 months before childbirth to 12 months postpartum were higher than those at 6 months before childbirth. Linear mixed models adjusting for possible confounding factors were also performed, but the significance of time points and courses of toenail cortisol levels were not significantly altered.

## Discussion

The present study investigates whether the expected changes in cortisol levels during pregnancy and after delivery are observed in nail cortisol and are accompanied by a time delay of several months owing to nail growth. A significant elevation in fingernail cortisol levels was found around childbirth, peaking at 2 months postpartum. These results were not altered by confounding factors. In addition, primiparas exhibited higher fingernail cortisol levels after childbirth. Furthermore, a significant elevation in toenail cortisol levels was found in the third trimester of pregnancy, and the levels remained elevated for 12 months postpartum. To our knowledge, this is the first study to investigate the time-course changes in nail cortisol levels during pregnancy and postpartum.

Cortisol levels in fingernail clippings increased from 1 month before childbirth to 4 months postpartum, peaking at 2 months postpartum. The time course of fingernail cortisol seemed to replicate the longitudinal change in cortisol levels in the body, as reported in previous studies^16,17^, with a time lag of several months. These results were not significantly altered by possible confounding factors such as age, BMI, seasons of nail collection, COVID-19 pandemic, postpartum depression, nail volume obtained in each collection, and daily use of soap, ethanol, and detergent. It should be noted, however, that this point needs to be tested on a larger sample. Previously, cortisol levels in fingernail clippings were retrospectively correlated with cortisol levels in hair and saliva samples, with a time lag of 2–5 months^14,15^. In addition to previous evidence, this study demonstrated evidence of intra-individual changes in cortisol levels in fingernail clippings in situations where cortisol levels in the body could be expected to change.

Some possible mechanisms for the accumulation of cortisol in the fingernails are as follows: passive diffusion from capillaries into the nail matrix is thought to be the main mechanism for the accumulation of cortisol in fingernails^8^. In addition to the matrix, the nail bed and external biological fluids (e.g., sweat) have been proposed as other possible mechanisms of drug incorporation into nails^21^. The nail bed is filled with blood vessels and contributes to nail growth; approximately 20% of the nail mass can be produced by the nail bed^22^. In a previous study^23^ that administered a single dose of the drug zolpidem resulted in three concentration peaks in fingernails: one after 24 h, one a few weeks, and one 3 months later, interpreted as corresponding to sweat-, nail bed-, and nail matrix-mediated drug incorporation, respectively. In the present study, the peak cortisol concentration in fingernail clippings was observed 2 months postpartum, implying the uptake of cortisol via the nail matrix. In addition, the nail bed could partially contribute to the elevation of fingernail cortisol levels around the time of delivery.

Compared with multiparas, primiparas exhibited higher cortisol levels in their fingernails. Primiparas also exhibited higher pregnancy-specific distress and higher serum cortisol levels during pregnancy among 137 women^18^. Furthermore, other studies investigating 24 h urinary^24^ and hair cortisol^19^ have reported higher cortisol levels in primiparas than in multiparas during late pregnancy. The results of the higher fingernail cortisol levels for primiparas were consistent with previous findings, which could support the biological plausibility of fingernail cortisol.

We also found a significant elevation in fingernail cortisol 8–9 months postpartum. In general, fingernails take 3–5 months to fully extend from the matrix^7^, and the elevation of 8–9 months postpartum might mirror the variations in cortisol in the body after giving birth. Previously, plasma cortisol levels have been shown to sharply decrease after childbirth (within 1 month), but the cortisol levels in the postpartum period were still higher than those in non-pregnant women^25^. In addition, the activity of the enzyme 11β-hydroxysteroid dehydrogenase type 1 (11β-HSD-1, which converts inactive cortisone to cortisol) peaked at 6 months postpartum^26^. However, the overall understanding of the hypothalamic–pituitary–adrenal activity for 1 year postpartum is insufficient. In this study, primiparas exhibited higher fingernail cortisol 8–9 months postpartum, but possible confounding factors, such as postpartum depression, were comparable between primiparas and multiparas (S1 Table). Unfortunately, we did not collect information on breastfeeding and the timing of resumption of menses after childbirth, and further studies are required to explore the factors associated with this cortisol elevation.

For toenail cortisol levels, we found a significant elevation in toenail clippings collected in the third trimester of pregnancy, and cortisol levels remained elevated for 12 months postpartum. The time course of toenail cortisol levels was significantly different from that of fingernail cortisol, which was unexpected as it takes approximately 8–14 months for toenails to fully extend from the matrix^7^. Unlike fingernail cortisol, the biological validity of toenail cortisol levels has not been thoroughly investigated. However, a previous study investigating the drug concentrations in toenails after single administrations of some pharmaceutical products observed higher concentrations of these pharmaceutical products in toenail clippings collected within 1 month after administration^27^. We speculate that sweat-mediated incorporation, in addition to nail matrix- and nail bed-mediated incorporation, could significantly contribute to cortisol levels in toenail samples because, compared to fingernails, toenails could be frequently exposed to sweat for longer periods in daily life (for example, while wearing socks and shoes). Therefore, the validity of toenail cortisol should be carefully interpreted because cortisol contained in toenail clippings could be contaminated by sweat.

This study has certain limitations that may have affected the interpretation of the findings. First, considering the high demands on the participants, that is, the nail collections over a long period (19 months), and the availability of financial resources, we included a relatively small number of participants. Second, certain confounding factors could have affected the time course of nail cortisol levels. For example, the period of this study partially overlapped with the COVID-19 pandemic, which caused changes in hygienic behaviors. Furthermore, pregnancy causes thicker nail plates^28^. Statistical adjustments of hygienic behaviors (daily use of soap and ethanol) or nail volume obtained in each collection did not alter the findings, but the effects of these confounding factors should be carefully considered. Third, we did not include a control group (non-pregnant women) in this study. In particular, toenail cortisol levels during the third trimester and postpartum period were relatively stable compared with fingernail cortisol levels, and comparison with non-pregnant women could shed light on the effect of pregnancy on toenail cortisol. Fourth, we did not collect blood or saliva samples and did not investigate the correlation of nail cortisol with blood or saliva cortisol because collection of blood or saliva samples for long periods, including the postpartum period, could increase the participants’ burden, yield poor adherence of the participants to the protocols, and result in them dropping out of the study.

In conclusion, in this study, the time courses of fingernail cortisol levels during pregnancy and postpartum were investigated. We observed that cortisol levels in fingernail clippings were elevated around childbirth, peaking at 2 months postpartum. In addition, variations in fingernail cortisol levels were associated with parity; primiparas exhibited higher fingernail cortisol levels after childbirth. These results imply that fingernail clipping might be a useful tool for monitoring cortisol variations in pregnant women, and could support the concept of nail cortisol levels as a retrospective index of hormone production.

## Methods

### Participants

In this 19-month observational study, 40 healthy pregnant women, less than 12 weeks of gestational age, were recruited from two maternity hospitals located in Yamaguchi Prefecture, Japan, from September 2018 to October 2019. The exclusion criteria were routine use of any medications (other than prenatal medications or supplements) at the time of enrollment, recent hormonal treatment, and smoking habits. Of the 40 participants, three dropped out and seven had multiple reports of steroid medication use (e.g., oral medications, ointments etc.) during the observation period, and were excluded from the data; finally, data from 30 women were analyzed.

### Statement of Ethics

This study was approved by the ethical committee of Nagaoka University of Technology (no. H30-1). All methods were carried out in accordance with the ethical guidelines determined by the National Ministry of Health, Labour and Welfare and the Declaration of Helsinki. Written informed consent was obtained from all the participants.

### Procedure

Fingernail and toenail samples were collected from the time of enrollment to 12 months postpartum. The study was conducted from September 2018 to May 2021. Participants were asked to collect their fingernails and toenails at intervals of 15 and 30 days, respectively; the collections were scheduled based on the estimated delivery date of each participant. They were instructed to provide nail samples from every digit by clipping the nail directly into a resealable polybag to avoid losing any part of the sample. They were also instructed not to use any manicures during the study period. On the day of nail collection, participants were reminded of the collection via e-mail and asked to report the completion of each collection on the online survey form. The samples were stored at room temperature and transported to the laboratory once per month.

Participants’ demographic information (age, height, weight, marital status, employment status, and history of giving birth) was obtained at the time of enrollment, and participants were checked for the use of medication and supplements once per month after enrollment. Furthermore, for the assessment of possible exposure of fingernails to chemical agents in daily life, such as surface-active agents and alcohol solvents, participants reported the frequency of washing hands using soap or alcohol (ethanol) and the use of detergent without wearing globes at three or 4 month-intervals during the study period (seven times in total). They also completed the Japanese version of the Edinburgh Postnatal Depression Scale (EPDS^29^;) once per month after giving birth (12 times in total). The EPDS is a screening test for postpartum depression consisting of 10 items, each scored from 0–3 points. The cutoff score was 8/9 for postpartum depression, and the sensitivity and specificity of this cutoff point are reported to be 0.75 and 0.93, respectively^29^.

### Measurements of fingernail cortisol levels

We followed the protocol of previous studies^14,30^ to determine the nail cortisol levels. Nail samples were washed twice in 5 ml of isopropanol and dried overnight. The samples were grounded using a cell disruptor (Multi Beads Shocker®; MB901RK, Yasui Kikai, Japan) at 2500 rpm for 2 min. Fifteen milligrams of nail powder were weighed, and 1.5 ml of pure methanol was added for cortisol extraction throughout 48 h under slow rotation. Following this, the samples were spun in a microcentrifuge at 10,000 rpm for 2 min, and 0.5 ml of the clear supernatant was evaporated at 60 °C until completely dry.

Cortisol levels of nail samples were determined by an enzyme immunoassay (EIA) method utilizing the EIA Kit (Salimetrics LLC, USA). The evaporated samples were resuspended in 50 μl of the assay diluent included in the EIA Kit, and the levels of cortisol in the diluent were analyzed according to the manufacturer’s instructions. For nail cortisol, the intra- and inter-assay variations were 5.5% and 4%, respectively. The findings were presented as pg cortisol/mg fingernails or toenails (pg/mg). The quantified levels of cortisol in finger and toe nails were all above the limit of quantification for EIA.

### Statistical analyses

For fingernail samples collected from 30 participants for 19 months (thus 1140 samples in the plan), two samples were not provided, and the cortisol levels for eight samples exceeded the range of the EIA kit. In addition, the cortisol levels for eight samples were considered outliers because they exceeded the mean + three standard deviations. One toenail cortisol sample was considered an outlier (570 samples in the plane) because it exceeded three standard deviations above the mean. Cortisol levels in fingernail and toenail samples were logarithmically transformed because the distribution was skewed. For fingernail cortisol, we calculated the average monthly cortisol level, in which the averages were weighted according to the fingernail volume obtained in each collection.

The variations in fingernail and toenail cortisol levels from 6 months before giving childbirth to 12 months postpartum were analyzed using linear mixed models with an unstructured error covariance matrix. Post-hoc comparisons were performed to compare cortisol levels at each time point with the baseline level (6 months before childbirth) using the Benjamini–Hochberg adjustment with a false discovery rate of 0.05. Considering the possible confounding factors, we also conducted linear mixed models adjusting for age, body mass index (BMI) before pregnancy, marital status, employment status, cesarean section during childbirth, season of nail collection (spring, summer, autumn, or winter), COVID-19 pandemic period (before or after the first declaration of a state of emergency due to COVID-19 in Japan), postpartum depression, nail volume obtained in each collection, frequency of washing hands using soap, and use of alcohol (ethanol), or frequency of detergent use. Each confounder was independently adjusted owing to the small sample size used in this study. Cortisol is a hormone that responds to stress. Possible confounding factors, such as marital and employment status, the COVID-19 pandemic, postpartum depression, and cesarean section were included. Also, some demographic and biological factors (age, BMI, seasons, nail volume) are reported to alter the growth rate and structure of nails^5^, and exposure of nails to daily life chemical agents (soap, ethanol, detergent) could alter the cortisol levels in nails. Therefore, we included these as confounding factors in the analyses.

Linear mixed models for fingernail cortisol were also used to investigate the interaction between time points and parity (primiparas vs. multiparas). These analyses were performed using the SPSS 27 software (IBM Corp., NY, USA). Statistical significance was set at *p* < 0.05.

### Supplementary Information

Supplementary Table S1.

## Data Availability

The datasets generated during and/or analysed during the current study are available from the corresponding author on reasonable request.

## References

[1] Dickerson SS, Kemeny ME (2004). Acute stressors and cortisol responses: A theoretical integration and synthesis of laboratory research. Psychol. Bull..

[2] Russell E, Koren G, Rieder M, Van Uum S (2012). Hair cortisol as a biological marker of chronic stress: Current status, future directions and unanswered questions. Psychoneuroendocrinology.

[3] Fischer S, Schumacher S, Skoluda N, Strahler J (2020). Fingernail cortisol - state of research and future directions. Front. Neuroendocrinol..

[4] Phillips R, Kraeuter AK, McDermott B, Lupien S, Sarnyai Z (2021). Human nail cortisol as a retrospective biomarker of chronic stress: A systematic review. Psychoneuroendocrinology.

[5] de Berker DA, André J, Baran R (2007). Nail biology and nail science. Int. J. Cosmet. Sci..

[6] Yaemsiri S, Hou N, Slining MM, He K (2010). Growth rate of human fingernails and toenails in healthy American young adults. J. Eur. Acad. Dermatol. Venereol..

[7] Shu I, Jones J, Jones M, Lewis D, Negrusz A (2015). Detection of drugs in nails: 3 year experience. J. Anal. Toxicol..

[8] Warnock F (2010). Measuring cortisol and DHEA in fingernails: A pilot study. Neuropsychiatr. Dis. Treat..

[9] Herane-Vives A (2018). Elevated fingernail cortisol levels in major depressive episodes. Psychoneuroendocrinology.

[10] Izawa S, Miki K, Tsuchiya M, Yamada H, Nagayama M (2019). Hair and fingernail cortisol and the onset of acute coronary syndrome in the middle-aged and elderly men. Psychoneuroendocrinology.

[11] Doan SN, DeYoung G, Fuller-Rowell TE, Liu C, Meyer J (2018). Investigating relations among stress, sleep and nail cortisol and DHEA. Stress.

[12] Izawa S, Matsudaira K, Miki K, Arisaka M, Tsuchiya M (2017). Psychosocial correlates of cortisol levels in fingernails among middle-aged workers. Stress.

[13] Wu H, Zhou K, Xu P, Xue J, Xu X, Liu L (2018). Associations of perceived stress with the present and subsequent cortisol levels in fingernails among medical students: A prospective pilot study. Psychol. Res. Behav. Manag..

[14] Izawa S (2015). Cortisol level measurements in fingernails as a retrospective index of hormone production. Psychoneuroendocrinology.

[15] Izawa S, Sugaya N, Ogawa N, Shirotsuki K, Nomura S (2021). A validation study on fingernail cortisol: Correlations with 1-month cortisol levels estimated by hair and saliva samples. Stress.

[16] Erickson K (2001). Preterm birth: Associated neuroendocrine, medical, and behavioral risk factors. J. Clin. Endocrinol. Metab..

[17] Sandman CA (2006). Elevated maternal cortisol early in pregnancy predicts third trimester levels of placental corticotropin releasing hormone (CRH): Priming the placental clock. Peptides.

[18] Gillespie SL, Mitchell AM, Kowalsky JM, Christian LM (2018). Maternal parity and perinatal cortisol adaptation: The role of pregnancy-specific distress and implications for postpartum mood. Psychoneuroendocrinology.

[19] Marteinsdottir I, Sydsjö G, Faresjö Å, Theodorsson E, Josefsson A (2021). Parity-related variation in cortisol concentrations in hair during pregnancy. BJOG.

[20] Frugé AD (2018). Fingernail and toenail clippings as a non-invasive measure of chronic cortisol levels in adult cancer survivors. Cancer Causes Control.

[21] Palmeri A, Pichini S, Pacifici R, Zuccaro P, Lopez A (2000). Drugs in nails: Physiology, pharmacokinetics and forensic toxicology. Clin. Pharmacokinet..

[22] Johnson M, Shuster S (1993). Continuous formation of nail along the bed. Br. J. Dermatol..

[23] Madry MM, Steuer AE, Binz TM, Baumgartner MR, Kraemer T (2014). Systematic investigation of the incorporation mechanisms of zolpidem in fingernails. Drug Test. Anal..

[24] Conde A, Figueiredo B (2014). 24-h urinary free cortisol from mid-pregnancy to 3-months postpartum: Gender and parity differences and effects. Psychoneuroendocrinology.

[25] Jung C (2011). A longitudinal study of plasma and urinary cortisol in pregnancy and postpartum. J. Clin. Endocrinol. Metab..

[26] Rogers SL, Hughes BA, Tomlinson JW, Blissett J (2016). Cortisol metabolism, postnatal depression and weight changes in the first 12 months postpartum. Clin. Endocrinol. (Oxford).

[27] Kuwayama K (2017). Time-course measurements of drug concentrations in hair and toenails after single administrations of pharmaceutical products. Drug Test. Anal..

[28] Altan Ferhatoğlu Z, Göktay F, Yaşar Ş, Aytekin S (2018). Morphology, growth rate, and thickness of the nail plate during the pregnancy. Int. J. Dermatol..

[29] Okano T (1996). Validation and reliability of Japanese version of EPDS (Edinburgh Postnatal Depression Scale). Arch. Psychiatr. Diagn. Clin. Eval..

[30] Izawa S, Yoshida R, Hasegawa-ohira M, Yamaguchi A, Nomura S (2016). Quantitative measurements of fingernail cortisol: Effects of ground-fingernail grain size and extraction time. Jpn. J. Physiol. Psychol. Psychophysiol..

