# Supplementary material for: Time-course changes in fingernail cortisol levels during pregnancy and postpartum
Source: Sci Rep. 2024 Jan 11;14:1145. doi: 10.1038/s41598-024-51650-2 (PMC10784579; doi:10.1038/s41598-024-51650-2)
Supplement: Supplementary file 1 — Supplementary Table S1. [file 41598_2024_51650_MOESM1_ESM.docx]

| Variables | Primiparas | Multiparas | P-value ^a)^ |
| --- | --- | --- | --- |
| Age (years) | 29.1 ± 5.1 | 32.7 ± 3.0 | 0.025 |
| BMI before pregnancy (kg/m^2^) | 20.6 ± 1.8 | 21.2 ± 3.8 | 0.064 |
| Marital status (married/unmarried) | 17/1 | 12/0 | 0.406 |
| Employment status (employee/unemployed) | 14/4 | 8/4 | 0.500 |
| Experience of cesarean section during childbirth (yes/no) | 1/17 | 1/11 | 0.765 |
| Season during childbirth (spring/summer/autumn/winter) | 5/3/5/5 | 4/1/5/2 | 0.742 |
| Giving birth during the COVID-19 pandemic (yes/no) | 3/15 | 1/11 | 0.511 |
| Experiencing depression during the 12 months postpartum (yes/no) ^b)^ | 10/8 | 4/8 | 0.232 |
| Washing hands using soap (frequency per day) ^c)^ | 8.8 ± 5.7 | 8.5 ± 4.8 | 0.898 |
| Washing hands using alcohol (frequency per day) ^c)^ | 2.4 ± 2.5 | 1.9 ± 1.3 | 0.054 |
| Detergent use without wearing gloves (frequency per day) ^c)^ | 3.4 ± 1.4 | 2.9 ± 1.1 | 0.650 |

**Supplementary Table 1.** Demographic data of primiparas (N = 18) and multiparas (N = 12).

a) *χ*^2^ tests and independent t-tests were conducted to compare the frequency or scores of the two groups.

b) Postpartum depression was considered experienced if participants exhibited a Japanese version of the Edinburgh Postnatal Depression Scale score ≥ 9 one or more times during the 12 months after childbirth.

c) Averages over the study period were demonstrated.
